# Economic evaluation of treatments for patients with localized prostate cancer in Europe: a systematic review

**DOI:** 10.1186/s12913-016-1781-z

**Published:** 2016-10-03

**Authors:** Virginia Becerra, Mónica Ávila, Jorge Jimenez, Laura Cortes-Sanabria, Yolanda Pardo, Olatz Garin, Angels Pont, Jordi Alonso, Francesc Cots, Montse Ferrer, on behalf of the Multicentric Spanish Group of Clinically Localized Prostate Cancer

**Affiliations:** 1 Health Services Research Group, IMIM (Hospital del Mar Medical Research Institute), Barcelona, Spain; 2 Universitat Pompeu Fabra, Barcelona, Spain; 3 CIBER en Epidemiología y Salud Pública, CIBERESP, Madrid, Spain; 4 Unidad de Investigación Médica en Enfermedades Renales, Hospital de Especialidades, IMSS, Guadalajara, México; 5 Epidemiology and Evaluation Department, IMIM (Hospital del Mar Medical Research Institute), Barcelona, Spain; 6 Health Services Research on Chronic Patients Network (Red de Investigación en Servicios de Salud en Enfermedades Crónicas [REDISSEC]), Barcelona, Spain; 7 Universitat Autònoma de Barcelona, Bellaterra, Spain

**Keywords:** Cost-effectiveness analysis, Cost-utility analysis, Cost-benefit analysis, Prostatic neoplasms, QALY, Quality-adjusted life years

## Abstract

**Background:**

Our objective was to assess the efficiency of treatments in patients with localized prostate cancer, by synthesizing available evidence from European economic evaluations through systematic review.

**Methods:**

Articles published 2000–2015 were searched in MEDLINE, EMBASE and NHS EED (Prospero protocol CRD42015022063). Two authors independently selected studies for inclusion and extracted the data. A third reviewer resolved discrepancies. We included European economic evaluations or cost comparison studies, of any modality of surgery or radiotherapy treatments, regardless the comparator/s. Drummond’s Checklist was used for quality assessment.

**Results:**

After reviewing 8,789 titles, 13 European eligible studies were included: eight cost-utility, two cost-effectiveness, one cost-minimization, and two cost-comparison analyses. Of them, five compared interventions with expectant management, four contrasted robotic with non robotic-assisted surgery, three assessed new modalities of radiotherapy, and three compared radical prostatectomy with brachytherapy. All but two studies scored ≥8 in the quality checklist. Considering scenario and comparator, three interventions were qualified as dominant strategies (active surveillance, robotic-assisted surgery and IMRT), and six were cost-effective (radical prostatectomy, robotic-assisted surgery, IMRT, proton therapy, brachytherapy, and 3DCRT). However, QALY gains in most of them were small. For interventions considered as dominant strategies, QALY gain was 0.013 for active surveillance over radical prostatectomy; and 0.007 for robotic-assisted over non-robotic techniques. The highest QALY gains were 0.57–0.86 for radical prostatectomy vs watchful waiting, and 0.72 for brachytherapy vs conventional radiotherapy.

**Conclusions:**

Currently, relevant treatment alternatives for localized prostate cancer are scarcely evaluated in Europe. Very limited available evidence supports the cost-effectiveness of radical prostatectomy over watchful waiting, brachytherapy over radical prostatectomy, and new treatment modalities over traditional procedures. Relevant disparities were detected among studies, mainly based on effectiveness. These apparently contradictory results may be reflecting the difficulty of interpreting small differences between treatments regarding QALY gains.

**Electronic supplementary material:**

The online version of this article (doi:10.1186/s12913-016-1781-z) contains supplementary material, which is available to authorized users.

## Background

Prostate cancer is the second most common cancer in men. An estimated 1.1 million men worldwide were diagnosed in 2012, with 345,000 cases in the European Union [1]. Estimates of public health expenditure on cancer indicate that prostate was the third contributor (6 % of the total), after colorectal and breast tumours [2]. Furthermore, United States (US) projections for the 2010–2020 period indicate a 27 % increase in cancer medical costs, where the largest is the continuing care phase of prostate cancer (42 %) [3].

Currently, most of the patients diagnosed (94 %) have localized prostate cancer [4] (ie, stage T1 or T2), and the number of treatments continues to increase [5, 6]. Despite the similar proven efficacy in terms of overall survival [7], these treatments differ substantially in their side effects pattern [8–11]. With so many different alternatives, health economics may contribute with relevant information for decision-making on treatment for localized prostate cancer [12], and there has been an increasing number of economic evaluations worldwide: comparing surgery versus radiotherapy [13, 14], different variations of prostatectomy [13, 15–17] or radiotherapy [13, 14, 18–21].

The National Institute for Clinical Excellence (NICE) published a global systematic review of economic evaluations for localized prostate cancer treatments in 2003 [22], before the new surgical and radiotherapy modalities appeared. Since, only two other systematic reviews have been published on economic evaluations. One, focusing on radiotherapy [23], identified 14 studies. The other one, evaluating radical prostatectomy, did not identify any complete economic evaluation meeting inclusion criteria, but instead included 11 cost comparison studies [24]. To our knowledge, there is no global systematic review that takes into account the economic evaluations of all treatments published during the last 15 years, including those comparing different therapies, such as radical prostatectomy versus radiotherapy or active surveillance. As a consequence, the efficiency of existing treatment options for localized prostate cancer is still uncertain.

Most of the economic evaluations were conducted in the US [23–26], yet differences in health systems across countries limit their results’ generalizability. Although there are also important differences within European countries, they share some major principles (such as a mainly publicly funded and almost universal coverage) far away from the insurance-based US health care system. Since economic evaluations are relevant to local context, our interest was centered in those performed in Europe. The aim of this study was to assess the efficiency of treatments in patients with localized prostate cancer, by synthesizing the available evidence from European economic evaluations through systematic review.

## Methods

The protocol was registered in PROSPERO (http://www.crd.york.ac.uk/Prospero) with number CRD42015022063. We conducted systematic searches in MEDLINE, EMBASE and NHS EED (NHS Economic Evaluation Database, CRD York) databases with a specific strategy (see online Additional file 1) from January 1st 2000 to December 31st 2015.

We looked for economic evaluations (cost minimization, cost-effectiveness, cost-utility, and cost-benefit analyses) or cost comparison studies that assessed any modality of surgery or radiotherapy treatments, regardless of the comparator/s, for patients with localized prostate cancer (T1–T2). Articles were considered when referring to any European country, and published in any European language.

Studies were excluded if they only performed cost estimations without comparing treatments (such as cost studies, cost of illness studies, or budget impact analyses); they were not primary studies (reviews, editorials or commentaries); they assessed patients with advanced prostate cancer; or they evaluated diagnosis or screening procedures, but no treatments.

Two members of the study team (JJ and VB) independently reviewed articles found in the literature search by examining them in three consecutive phases: titles, abstracts, and full text. A third reviewer (MA) resolved discrepancies. A pilot test was performed to homogenize criteria among reviewers. Finally, the reference lists of the selected articles and those of previous systematic reviews were reviewed to identify other possible studies that could be included. Coding for inclusion and exclusion criteria were defined and recorded for each stage.

Assessment of studies’ quality and data extraction was performed by the consensus of two reviewers (VB and MA). Drummond’s Checklist was used for quality assessment [27]. Data was extracted using a standardized, pre-piloted data collection form, including participant characteristics, interventions, comparator, economic perspective, and time horizon among others. The pre-defined primary outcome to be extracted was the incremental cost per Quality-Adjusted Life-Year (QALY) gained. Other Incremental Cost-Effectiveness Ratios (ICERs) and comparative costs per treatment were considered secondary outcomes. For illustrative purposes a figure has been designed to show all estimations of accumulated cost converted into euros (considering the current 2015 exchange rates), and plotted them through the time horizon for each intervention. Patient Intervention Comparator Outcome (PICO) strategy for this review is shown in the online Additional file 2.

## Results

### Literature flow in the systematic review

**Fig. 1:**
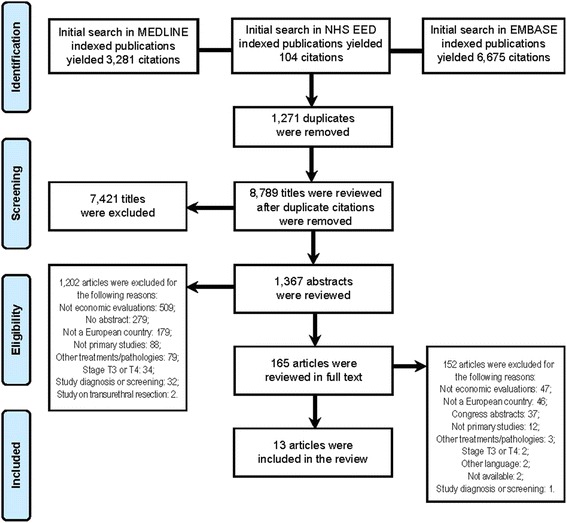
Preferred Reporting Items for Systematic Reviews and Meta-Analyses (PRISMA) Flow of Literature Diagram

Figure 1 shows the Preferred Reporting Items for Systematic Reviews and Meta-Analyses (PRISMA) diagram. Once 1,271 duplicates were excluded, 8,789 titles and 1,367 abstracts were reviewed, 165 articles were fully read, and finally only 13 eligible studies were included. Overall agreement and kappa coefficients (k) between reviewers were 79.7 % (*k* = 0.35), 92.8 % (*k* = 0.63), and 88.3 % (*k* = 0.53) in the title, abstract, and full text stages, respectively.

### Characteristics of economic evaluations identified in the systematic review

Table 1 shows the characteristics of the 13 economic evaluations which met the inclusion criteria [22, 28–39]. Most were conducted in the United Kingdom (UK), Sweden, and France. All were complete economic evaluations, except two cost-comparisons [30, 34]: eight were cost-utility analyses, two cost-effectiveness analyses [31, 39] and one cost-minimization analysis [38]. Studies were classified according to the treatments they evaluated: a) in five studies [22, 28–31] interventions were compared with expectant management (watchful waiting or active surveillance); b) four studies compared robotic-assisted laparoscopic prostatectomy with other surgical techniques [32–35]; c) three studies contrasted conventional external radiotherapy with new modalities [22, 36, 37] (Intensity-Modulated Radiation Therapy–IMRT, proton therapy and brachytherapy); and d) three studies compared radical prostatectomy with radiotherapy [22, 38, 39]. Only the 2003 Hummel et al. study [22] provided data for more than one of these classification groups (a, c and d).

**Table 1:** Characteristics of economic evaluations identified in the systematic review

Authors (Year) Country [Reference]	Population	Interventions (No. patients)	Economic Perspective (Time Horizon)	Source for Costs data (year)	Source for Effects data	Type of Evaluation (Design/Model) [Threshold for Cost-Effectiveness]
A. Expectant management (active surveillance or watchful waiting) vs other treatments						
Koerber, et al. (2014) Germany [28]	Theoretical cohort Mean 65 Years LE > 15 years PSA ≤10 ng/ml Gleason: ≤6 Stage:≤T2a No severe comorbidities	AS RP (No. patients Not applicable)	Societal (Lifetime)	Published literature German DRG, physician’s fee, pharmaceutical prices catalogues (2011) Discount rate 3 %	Disease mortality from SCPG-4 data Baseline utilities: German survey with EQ-5D Health state specific utilities: published literature	Cost-utility analysis (Markov model) [€50,000 per QALY gained]
Lyth, et al. (2012) Sweden [29]	Randomized trial SPCG-4 Age < 75 years LE > 10 years PSA < 50 ng/ml No other cancer	WW RP (n total = 695)	Payer (10 years)	Retrospectively collected in SPCG-4 trial patient records. (2007) Discount rate 3.5 %	Individual-patients data from SPCG-4 with a 77-item questionnaire	Cost-utility analysis (Semi-Markov model) [200,000 SEK per QALY gained]
Andersson, et al. (2011), Sweden [30]	Randomized trial SPCG-4 Age < 75 years LE > 10 years PSA < 50 ng/ml	WW (*n* = 105) RP (*n* = 107)	Payer (12 years)	Medical records and price list at the University Hospital in Örebro (2007)	NA	Cost Comparison (Not modelling) [Not Applicable]
Bauvin, et al. (2003) France [31]	Retrospective control-cohort study (patients diagnosed in 1995)	WW (*n* = 46) RP (*n* = 56)	Payer (5 years)	Delphi method (1995) Discount rate 3 %	Survival at 5 years from individual-patients data	Cost-effectiveness analysis (Not modelling) [Not Reported]
Hummel, et al. (2003) UK [22]	Theoretical cohort Age: 65-year old	WW BT 3DCRT	Payer (Lifetime)	Literature review and NHS trusts (2002) Discount rate 6 %	Literature review for Utilities Authors assume equal disease-free survival effectiveness	Cost-utility (Markov model) [£20,000 and £30,000 per QALY gained]
B. Robot-assisted laparoscopic prostatectomy (RALP) vs other surgical techniques						
Lord, et al. (2013) UK [32]	Theoretical cohort	RRP (*n* = 1000) PRP (*n* = 1000) LRP (*n* = 1000)) RALP (*n* = 1000)	Payer (Lifetime)	NHS data & Literature review. (2010–11) Discount Rate 3.5 %	Disease registries and recent UK systematic reviews and meta-analyses.	Cost-utility analysis (Individual-level Discrete event simulation) [£20,000 per QALY gained]
Close, et al. (2013) UK [33]	Theoretical cohort Mean 61.5 years	RALP (*n* = 5000) LRP (*n* = 5000)	Payer (10 years)	UK NHS da Vincy Surgical System prices provided by the manufacturer. (2009) Discount rate: 3.5 %	Systematic literature review and meta-analysis of clinical effectiveness and expert advisory group	Cost-utility analysis (Discrete event simulation model) [£30,000 per QALY gained in base case £0 to £50,000 in Sensitivity Analysis]
Barbaro, et al. (2012) Italy [34]	Observational prospective cohort study Treatment 2007–8 Mean 63.8 years	RRP (*n* = 99) RALP (*n* = 24)	Hospital (hospital stay)	Patient’s medical health record and operating room report. Hospital accounting office reimbursement fees. (2008)	Primary data from the study itself	Cost Comparison [Not Applicable]
Hohwu, et al. (2011) Denmark [35]	Retrospective cohort Age: 50–69 years Treatment 2004-7	RALP (*n* = 77) RRP (*n* = 154)	Societal (1 year)	Medical records, price list hospital and national registries. Absence from work using the human capital method. (2008)	Primary data from the study itself SF-6D from SF-36 questionnaire	Cost-utility analysis [Not Reported]
C. Conventional external radiotherapy vs new modalities						
Hummel, et al. (2012) UK [36]	Theoretical cohort Age 70 years	IMRT 3DCRT (10000 patients for each model)	Payer (Lifetime)	St Bartholomew’s hospital Literature review, expert opinion. None primary data collected on resource use. (2008) Discount rate 3.5 %	Systematic literature review	Cost-utility analysis (Discrete event simulation model) [£20000 and £30000 per QALY gained]
Lundkvist, et al. (2005) Sweden [37]	Theoretical cohort Age: 65-year	PT External Radiotherapy	Payer (Lifetime)	Published literature and assumptions (2002) Discount rate 3 %	Published literature	Cost-utility analysis (Markov model) [€55000 per QALY gained]
Hummel, et al. (2003) UK [22]	Theoretical cohort Age: 65-year old	2DRT BT 3DCRT	Payer (Lifetime)	Literature review and NHS trusts. (2002) Discount rate 6 %	Literature review for Utilities Authors assume equal disease-free survival effectiveness	Cost-utility (Markov model) [£20000 and £30000 per QALY gained]
D. Prostatectomy vs radiation treatment						
Becerra, et al. (2011) Spain [38]	Observational prospective cohort Mean age: RP = 63.7 years BT = 67.6 years 3DCRT = 69 years	RP (*n* = 181) BT (*n* = 64) 3DCRT (*n* = 153)	Payer (6 months)	Micro costing from reference hospitals, patient charts, tariffs and previously published data. (2004–2005). Not discount rate	Equally effective	Cost minimization (Not modelling) [Not Applicable]
Buron, et al. (2007) France [39]	Observational retrospective cohort 11hospitals PSA ≤20 ng/ml Gleason < 8.	RP (*n* = 127) BT (*n* = 308)	Societal (2 years)	French National Security fee schedule for DRG and outpatient. Production loss: French daily national average wage. (2001)	EORTC core QLQ-C30 and EORTC QLQ-PR25.	Cost-effectiveness analysis (Not modelling) [Not Reported]
Hummel, et al. (2003) UK [22]	Theoretical cohort Age: 65-year old	RP BT 3DCRT	Payer (Lifetime)	Literature review and NHS trusts. (2002) Discount rate 6 %	Literature review for Utilities Authors assume equal disease-free survival effectiveness	Cost-utility (Markov model) [£20000 and £30000 per QALY gained]

*Abbreviations*: *BT* Brachytherapy, *DRG* Diagnosis Related Group, *SPCG-4 trial* Scandinavian Prostate Cancer Group Study Number 4 trial, *AS* Active Surveillance, *IMRT* Intensity-Modulated Radiation Therapy, *LE* Life Expectancy, *LRP* Laparoscopic Prostatectomy, *RALP* Robot-Assisted Laparoscopic Prostatectomy, *RP* Radical Prostatectomy, *PRP* Perineal Radical Prostatectomy, *RRP* Radical Retropubic Prostatectomy, *PR* Proton therapy, *PSA* Prostate Specific Antigen, *QALYs* Quality-Adjusted Life Years, *WW* Watchful Waiting, *2DRT* Two Dimensional Radiotherapy, *3DCRT* Three Dimensional Conformal Radiotherapy

Most of the evaluations (nine out of 13) were conducted from a payer’s perspective. Regarding the time horizon, lifetime (assuming an age limit of 100 years) was considered in five studies [22, 28, 32, 36, 37], one decade in three other studies [29, 30, 33], and shorter periods for the rest (from hospital stay to 5 years). Source of cost was medical records from study cohorts, such as the Scandinavian Prostatic Cancer Group Study Number 4 (SPCG-4) [40], or national database registers of activities such as the British National Health System (NHS) or, more rarely, only literature review (two studies) [36, 37]. Similar sources were used for effects on health. Only in seven studies the threshold to consider an alternative as cost-effective was clearly stated [28, 29, 32, 33, 36, 37, 41]. It ranged from €20,000 to €55,000 per QALY gained, and four studies carried out sensitivity analysis around this threshold [22, 28, 32, 33].

### Main findings of economic evaluations identified in the systematic review

Estimated total direct cost for every treatment alternative was reported in all but two of the studies (see Table 2), which only showed incremental cost difference [29, 37]. Eight studies could provide incremental cost per QALY gained [22, 28, 29, 32, 33, 35–37], and four studies other outcomes such as life year gained [28], 5-year survival [31], successful treatment [35], and treatment side effects [39].

**Table 2:** Main findings of economic evaluations identified in the systematic review

Authors (Year) Country [Reference]	Mean Cost Mean Incremental (Δ) Cost	Effectiveness measure or Incremental (Δ) QALYs	ICER	Sensitivity Analyses	Conclusions
A. Expectant management (active survaillance or watchful waiting) vs other treatments					
Koerber, et al. (2014) [28]	Mean Cost: RP €16468; AS €9585 Mean Δ Cost RP vs AS: €6883	Life expectancy: RP 12.15; AS 12.07 QALYs: RP 7.56; AS 7.60	€/Life year gained for RP: 96420 €/QALY gained: AS resulted a dominant strategy over RP.	-Probability of metastases in AS -AS utility weights -Time horizon: 5, 15 and 30 years. -Discount rate 0,5,7 and 10 %	“AS is likely to be a cost-saving treatment strategy for some patients with early stage localized prostate cancer. However, cost-effectiveness is dependent on patients’ valuation of health states […]”
Lyth, et al. (2012) [29]	Mean Δ Cost RP vs WW: S1-SEK 40116 S2-SEK 49784 S3-SEK 59160 S4-SEK 63834 S5-SEK 70074 S6-SEK 72439	Δ QALY: S1-0.57 S2-0.86 S3-0.25 S4-0.42 S5-0.08 S6-0.15	SEK/QALY gained for RP: S1-70766 S2-58045 S3-232409 S4-150274 S5-858703 S6-472327	Scenarios: S1-65y Gleason 0–4 S2-65y Gleason 5–6 S3-70y Gleason 0–4 S4-70y Gleason 5–6 S5-75y Gleason 0–4 S6-75y Gleason 5–6	“Assuming a threshold value of 200000 SEK/QALY gained, for patients aged ≤70 years the treatment is always cost-effective, except at age 70, Gleason 0–4 and PSA ≤10 […]”
Andersson, et al. (2011) [30]	Mean Cost: RP €24247; WW €18124	Not Applicable	Not Applicable	Not Applicable	“In this economic evaluation of RP versus WW of localized prostate cancer in a randomized study, RP was associated with 34 % higher costs. […]”
Bauvin, et al. (2003) [31]	Mean Cost: RP €8533; WW €2143	5 year survival: RP 89 %; WW 78 % 5 year relative survival: RP 97 %; WW 95 %	ICER not reported	Not reported	Results supported the cost-effectiveness of radical prostatectomy over watchful waiting.
Hummel, et al. (2003) [22]	Mean Cost: WW £1714 BT £6880 3DCRT £2103	QALYs: WW 8.88 BT 9.28 3DCRT 8.89	£/QALY gained (WW as reference): -12828 for BT -26766 for 3DCRT	-Incidence of adverse events -Utilities -Age -Costs	“[…] It is difficult therefore to draw conclusions on the relative benefits or otherwise of the newer technologies owing to the lack of substantive evidence of any quality and the lack of comparisons between the newer technologies and with standard treatments. […]”
B. Robot-assisted laparoscopic prostatectomy (RALP) vs other surgical techniques					
Lord, et al. (2013) [32]	Mean Costs: RRP £6485; LRP £6534 PRP £6510; RALP £6458	QALYs: RRP 7.937; LRP 7.936 PRP 7.936; RALRP 7.943	£/QALY gained: RALP resulted a dominant strategy over all other	-Willingness-to-pay threshold	“[…] The practical usefulness of our models to guideline developers and users should also be investigated, as should the feasibility and usefulness of whole guideline modelling alongside development of a new Clinical Guidelines.”
Close, et al. (2013) [33]	Mean Costs: RALP £9040; LRP £7628 N° Procedures/year (P/year) 200 RALP £9040; LRP £7628 150 RALP £9799; LRP £7628 100 RALP £11312; LRP £7628 50 RALP £15859; LRP£7628 Three-arm robot (Da Vinci®) with 200 P/year: RALP £8168; LRP £7628	QALYs: RALP 6.52; RLP 6.44	£/QALY gained for RALP: -18329 for 200 P/year -28172 for 150 P/year -47822 for 100 P/year -106839 for 50 P/year Three-arm robot (DaVinci®)) £7009/QALY for 200 P/year	-Positive margin rate after RALP -Procedures/year -Patient’s lifetime -Price of robotic system	“Higher costs of robotic prostatectomy may be offset by modest health gain resulting from lower risk of early harms and positive margin, provided >150 cases are performed each year. Considerable uncertainty persists in the absence of directly comparative randomised data.”
Barbaro, et al. (2012) [34]	Mean Surgical Costs: RALP €20103; RRP €2764 Mean Hospital Costs: RALP €3358; RRP €2791 Mean Total Costs: RALP €23610; RRP €5635	Not Applicable	Not Applicable	-Case volumes -Operating times	” In the current circumstances, increasing the use of RAP at the San Giovanni Battista Hospital does not appear expedient. This conclusion is corroborated by the sensitivity analysis which showed that RAP carries higher costs than RRP.”
Hohwu, et al. (2011) [35]	Mean direct costs: RALP €8369 RRP €3863 Mean Indirect costs: RALP €13411 RRP €12465	Successful treatment: RALP 34 %; RRP 27 % Δ QALYs: RALP 0.0103; RRP 0.0116	€/extra successful treatment for RALP -64343 for direct costs -13514 for indirect costs €/QALY gained for RALP: Not applicable because no QALY gained	-Life time for robot -Procedures/year	“RALP was more effective and more costly. A way to improve the cost effectiveness may be to perform RALP at fewer high volume urology centres and utilise the full potential of each robot”
C. Conventional external radiotherapy vs new modalities					
Hummel, et al. (2012) [36]	Mean total discounted costs: IMRT/3DCRT S1-£6173/£5184 S2-£4946/£4214 S3-£4946/£4486 S4-£5687/£7489	Total discounted QALY: IMRT/3DCRT S1-6.802/6.792 S2-7.070/7.046 S3-7.070/6.983 S4-7.015/6.402	£/QALY gained for IMRT: S1-104066 S2-31162 S3-5295 S4-dominant strategy.	Scenarios: S1-equal dose& PSA relapse S2-15 % difference in late gastro intestinal toxicity S3-3.8 y survival difference S4-6.6 y survival difference	“If IMRT can be used to prolong survival, it is very cost-effective. Otherwise cost-effectiveness is uncertain”
Lundkvist, et al. (2005) [37]	Δ total cost for standard case Proton Therapy vs External Radiotherapy: €7953 per patient,	Δ QALY for Proton Therapy: 0.297/patient	€/QALY gained for Proton Therapy:–26776	Not reported	“Proton therapy was cost-effective if appropriate risk groups were chosen. The results must be interpreted with caution, since there is a lack of data, and consequently large uncertainties in the assumptions used”
Hummel, et al. (2003) [22]	Mean total costs: 2DRT £1886 BT £6880 3DCRT £2103	QALYs: 2DRT 8.56 BT 9.28 3DCRT 8.89	£/QALY gained (2DRT as reference): -8575 for BT -683 for 3DCRT	-Incidence of adverse events -Utilities -Age -Costs	See above
D. Prostatectomy vs radiation treatment					
Becerra, et al. (2011) [38]	Mean total cost: RP €6863.70 BT €5453.60 3DCRT €3336.10	Not Applicable	Not Applicable	-Cost of 3DCRT	“Radical prostatectomy therapeutic proved to be the most expensive treatment option. […] Most of the costs were explained by the therapeutic option, and neither comorbidity nor risk groups showed an effect of total costs independent of treatment.”
Buron, et al. (2007) [39]	Mean societal cost: BT €8019; RP €8715 Mean Initial treatment costs: BT €7159; RP €6472 Mean hospital follow-up costs: BT €268; RP €992 Mean Outpatient costs: BT €482; RP €419 Mean loss productivity costs: BT €620; RP €3678	Urinary incontinence BT 20 %; RP 49 % Fecal incontinence BT 9 %; RP 2 % Rectal Bleeding BT 15 %; RP 0 % Erectile Dysfunction BT 45.8 %; RP 83.3 %	ICER not reported	Not reported	“This study suggests a similar cost profile in France for BT and RP but with different health-related quality of life and side effect profiles. Those findings may be used to tailor localized prostate cancer treatments to suit individual patients’ needs.”
Hummel, et al. (2003) [22]	Mean total costs: RP £6359 BT £6880 3DCRT £2103	QALYs RP 8.93 BT 9.28 3DCRT 8.89	£/QALY gained (RP as reference): -12828 for BT -Not Applicable	- Incidence of adverse events -Utilities -Age -Costs	See above

*Abbreviations*: *AS* Active Surveillance, *BT* Brachytherapy, *ICER* Incremental Cost-Effectiveness Ratio, *IMRT* Intensity-Modulated Radiation Therapy, *LRP* Laparoscopic Prostatectomy, *RALP* Robot-Assisted Laparoscopic Prostatectomy, *RP* Radical Prostatectomy, *PRP* Perineal Radical Prostatectomy, *RRP* Radical Retropubic Prostatectomy, *QALYs* Quality-Adjusted Life Years, *WW* Watchful Waiting, *2DRT* Two Dimensional Radiotherapy, *3DCRT* Three Dimensional Conformal Radiotherapy

Of the interventions evaluated, three were found to be not only cost-effective but also dominant strategies (more effective and less costly): active surveillance over radical prostatectomy from a societal perspective in Germany [28], robotic-assisted over non-robotic surgical techniques [32], and IMRT over 3-Dimensional Conformal Radiation Therapy (3DCRT) when assuming a survival improvement of 6.6 years [36]. The following six interventions were found to be cost-effective: radical prostatectomy over watchful waiting in patients aged 70 or younger [29], robotic-assisted over non-robotic laparoscopic radical prostatectomy if more than 150 procedures performed per year [33], IMRT over 3DCRT when survival improvement is ≥3.8 years [36], and proton therapy [37], brachytherapy [22] and 3DCRT [22] over conventional radiotherapy. Conversely, the highest cost per QALY gained (least efficient options) were shown for radical prostatectomy versus watchful waiting in patients older than 75 [29], robotic-assisted versus non-robotic radical prostatectomy performing 50 procedures per year [33] (over £100,000), and for IMRT versus 3DCRT at equal doses and same survival to Prostate-Specific Antigen (PSA) progression [36] (over €100,000).

**Fig. 2:**
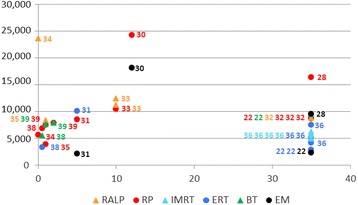
Estimations of accumulated direct costs (euros) for each intervention plotted through the time horizon (years). Numbers correspond to the articles in the reference list. Abbreviations: RALP: robot-assisted laparoscopic prostatectomy; RP: radical prostatectomy; IMRT: intensity-modulated radiation therapy; ERT: external radiation therapy; BT: brachytherapy; EM: expectant management

Estimations of accumulated direct costs in euros were plotted through the time horizon in Fig. 2 for each intervention. In total, the figure shows 38 estimates reported by 11 studies. The lowest costs (around €2,000) were obtained for expectant management (specifically, watchful waiting) at time horizons of 5 years and lifetime, as reported by Bauvin et al. [31] and Hummel et al. [22], respectively. The highest costs (around €24,000) were obtained for robotic-assisted surgery during hospitalization [34] and for radical prostatectomy at 12 years [30].

### Quality of the economic evaluations identified in the systematic review

The quality of the studies according to Drummond’s 10-item checklist is illustrated in Table 3. From the 11 economic evaluations, nine studies scored ≥8 points. The item that most frequently failed was about effectiveness, appraised uncertain or negative in six studies.

**Table 3:** Methodological quality assessment of economic evaluations using Drummond’s 10-item checklist

(Yes/no/can’t tell)	Koerber [28]	Lyth [29]	Bauvin [31]	Hummel [22]	Lord [32]	Close [33]	Hohwu [35]	Hummel [36]	Lundkvist [37]	Becerra [38]	Buron [39]
1. Was a well-defined question posed in answerable form?	Yes	Yes	Yes	Yes	Yes	Yes	Yes	Yes	Yes	Yes	Yes
2. Was a comprehensive description of the competing alternatives given (i.e. can you tell who did what to whom, where, and how often)?	Yes	Yes	Yes	Yes	Yes	Yes	Yes	Yes	Yes	Yes	Yes
3. Was the effectiveness of the programme or services established?	Can’t Tell	Can’t Tell	Yes	No	Can’t Tell	Yes	Yes	Yes	Can’t Tell	No	Yes
4. Were all the important and relevant costs and consequences for each alternative identified?	Yes	Yes	Yes	Yes	Yes	Yes	Yes	Yes	Can’t Tell	Yes	Yes
5. Were costs and consequences measured accurately in appropriate physical units’	Yes	Yes	No	Yes	Yes	Yes	Yes	Yes	Can’t Tell	Yes	Yes
6. Were costs and consequences valued credibly?	Yes	Yes	Can’t Tell	Yes	Yes	Yes	No	Yes	Can’t Tell	Yes	Yes
7. Were costs and consequences adjusted for differential timing?	Yes	Yes	Yes	Yes	Yes	Yes	No	Yes	Yes	No	No
8. Was an incremental analysis of costs and consequences of alternatives performed?	Yes	Yes	No	Yes	Yes	Yes	Yes	Yes	Yes	Yes	Yes
9. Was allowance made for uncertainty in the estimates of costs and consequences?	Yes	Yes	No	Yes	Yes	Yes	Yes	Yes	No	Yes	Yes
10. Did the presentation and discussion of study results include all issues of concern to users?	Yes	Yes	Can’t Tell	Yes	Yes	Yes	Yes	Yes	Can’t Tell	Yes	Yes
Score (Total)	9	9	5	9	9	10	8	10	4	8	9

Number between square brackets corresponds to reference list position

## Discussion

Our systematic literature review identified 13 European studies, published 2000–2015, which conducted either economic evaluations or cost comparisons (11 and two, respectively) of any modality of surgical or radiotherapy treatments for localized prostate cancer patients. These studies varied widely in compared alternatives, costing methodologies, and time horizon. Estimations of incremental cost per QALY gained were provided by eight studies. Depending on the scenario and the comparator considered, three interventions were qualified as dominant (active surveillance [28], robotic-assisted surgery [32], and IMRT [36]), and six as cost-effective (radical prostatectomy [29], robotic-assisted surgery [33], IMRT [36], proton therapy [37], brachytherapy [22] and 3DCRT [22]).

### Expectant management (active surveillance or watchful waiting) vs other treatments

Two cost-utility analyses comparing radical prostatectomy with expectant management show contradictory results: Koerber et al. [28] found that active surveillance was the dominant alternative (more QALYs at less cost), while Lyth et al. [29] showed that radical prostatectomy was more cost-effective than watchful waiting. However, the gain in QALYs in favor of active surveillance was extremely small (0.013) [28], and moderate-to-small in favor of radical prostatectomy (0.57–0.86) [29]. On the other hand, differences in the comparator used in both studies (active surveillance [28] and watchful waiting [29]) could also partly explain this disparity. No immediate treatment was performed in watchful waiting patients [29], while active surveillance involved [28] monitoring with PSA, digital rectal examination, and biopsy. Consistent with results reported by Lyth et al. [29], the cost-effectiveness study by Bauvin et al. [31] showed that radical prostatectomy is more effective than watchful waiting. Unfortunately, although the economic evaluation of Hummel et al. [22] also evaluated radical prostatectomy, they did not report its comparison with watchful waiting.

### Robot-assisted laparoscopic prostatectomy (RALP) vs other surgical techniques

The previous systematic review of economic evaluations comparing robotic-assisted vs non-robotic laparoscopic surgery [24] proved to be insufficient for decision making, leading the authors to build a de novo economic evaluation [33], which has been now included in our review. Two of the three cost-utility studies that we identified consistently support the cost-effectiveness of robotic-assisted surgery [32, 33]. Lord et al. [32] showed that robotic-assisted technique is the dominant alternative among surgery, while Close et al. [33] estimated a cost of £18,329 per QALY gained. Hohwu et al. [35] found no QALY gain for robotic-assisted surgery, but the authors underlined the uncertainty of their QALY estimates due to a high degree of missing data. Again, disparity among these economic evaluations is mainly due to contradictory results on effectiveness, which were based on extremely small QALY gains for the robotic-assisted technique: 0.007 reported by Lord et al. [32], and 0.08 by Close et al. [33] In fact, current guidelines of the European Association of Urology [5, 6] consider all approaches (i.e., open, laparoscopic, and robotic) as acceptable for patients who are surgical candidates, because no single modality has shown a clear superiority in terms of functional or oncological results. On the other hand, it is important to highlight that the recommendation of the NICE Clinical Guideline [42] to provide robots in centers with an expected performance of at least 150 robotic-assisted operations per year, is only based on the economic evaluation published by Close et al. [33] It would be advisable to confirm this recommendation with future specific studies to help decision makers.

### Conventional external radiotherapy vs new modalities

The systematic review of cost-effectiveness analysis by Amin et al. [23], comparing different radiation treatments, identified 14 studies (most from the United States, and only two from Europe [22, 36]). Although evidence suggested that brachytherapy and IMRT were more cost-effective than external beam radiotherapy, the authors highlighted the uncertainties and variation among studies [23]. We only identified three European economic evaluations comparing radiation therapies, each focusing on a different new modality (IMRT [36], proton therapy [37], and brachytherapy [22]). The three showed to be more cost-effective than conventional radiotherapy. However, each of these findings came from only one study, so further research is needed to confirm them. Once again, it is important to point out that the magnitude of the QALY gains is small for scenarios evaluating IMRT (0.01–0.613) [36] or proton therapy (0.297) [37], and moderate-to-small in favor of brachytherapy (0.72) [22]. The European Association of Urology guidelines (5) recommend IMRT for definitive treatment with external radiotherapy, and brachytherapy for patients fulfilling specific criteria (low risk, prostate volume below 50 mL, no urinary obstruction, and no previous transurethral resection).

### Prostatectomy vs radiation treatment

Of the three studies comparing prostatectomy with radiation treatment, only Hummel et al. [22] published a cost-utility analysis showing that brachytherapy was more cost-effective than surgery, with an incremental cost of €2,021–2,760 per QALY gained. Buron et al. [39] did not calculate ICERs but showed similar societal costs between radical prostatectomy and brachytherapy, though different treatment side effects: radical prostatectomy caused higher rates of urinary incontinence and erectile dysfunction, while brachytherapy presented irritative urinary and bowel symptoms more frequently. These results are consistent with the well-known side effect profiles of these treatments [8–11]. The cost-minimization published by Becerra et al. [38] assumed equal effectiveness in terms of survival, but did not take into account other relevant outcomes such as relapses and treatment side effects. Thus, evidence supporting the cost-effectiveness of brachytherapy over open radical prostatectomy originates from one single study [22] showing a small QALY gain (0.35), and there are no economic evaluations comparing brachytherapy with robotic-assisted surgery.

### Accumulated direct costs per treatment

As shown in Fig. 2, the cost-comparison study performed in Sweden reported the highest estimation of costs for radical prostatectomy and watchful waiting (€24,247 and €18,124) [30]; also, the cost-comparison study published by Barbaro et al. [34] showed an extreme perioperative cost in an Italian hospital for robotic surgery (€23,610). The high cost estimated in these two empirical cost-comparison studies [30, 34] (based on the observation of health care activities in real cohorts) could indicate underestimation of real costs when they are based on models from theoretical cohorts. Furthermore, the surprisingly low accumulated costs estimated in most studies with theoretical cohorts and lifetime horizon [22, 32, 36], similar or even lower than those reported for studies with a shorter time horizon [31, 33], also suggest an underestimation of real costs in these studies.

### Cost and effectiveness components

Economic evaluations have two components. Regarding the cost component, it is important to highlight the similarities of the new treatment modalities compared with the traditional techniques, such as robotic versus non-robotic surgery [33] and IMRT versus external beam radiotherapy [36], when provided under rational conditions. Besides watchful waiting, the cheapest, all other treatments seem to be quite similar: most have an equivalent total cost below €17,000. The European estimates of accumulated direct healthcare costs identified are much lower than those reported in US. For instance, Cooperberg et al. [13] considering lifetime, and Hayes et al. with a 10 year horizon [14] reported costs figures of: $20,000–38,000 in radical prostatectomy; around $33,000 in 3DCRT; $38,000–54,000 in IMRT; or $25,000–44,000 in brachytherapy. Different health systems and cost structures between US and Europe may explain these variances.

Effectiveness is the most relevant component. However, the aforementioned disparities among studies in the identification of the most effective treatment may reflect the misinterpretation of such small QALY gains showed by the majority of them. For example, the gain of 0.013 QALYs [28] was much too small to consider active surveillance the dominant strategy over radical prostatectomy; or the gain of 0.007 QALYs [32] to consider robotic-assisted the dominant strategy over non-robotic techniques. Even the clinical relevance of the highest QALY gains identified in this review (0.57–0.86 for radical prostatectomy vs watchful waiting [29], and 0.72 for brachytherapy vs conventional radiotherapy [22]) may be questionable to be interpreted as relevant differences on effectiveness. Which is the reasonable cut-off for considering one intervention more effective than its alternative? Could gains lower than one QALY through 10 years or lifetime be considered clinically significant?

Results from US economic evaluations [13, 14] also showed no relevant differences in QALY gains for lifetime across treatments: ranging 0.5–1 or 0.7–0.8 for patients at low and intermediate risk, respectively, when comparing surgical and radiation therapies [13]; 0.9, 0.9, and 1.1 when comparing brachytherapy, IMRT and surgery with watchful waiting [14]. The clinical relevance of less than 1 year benefits between alternatives (in time horizons > 10 years of life) is questionable, and common sense prevents from interpreting them as differences in effectiveness.

An important issue related to the generalizability of study findings is the cost-effectiveness threshold, which represents society’s willingness-to-pay for an additional unit of benefit [26]. Studies from UK showed a very consistent pattern regarding this threshold: they considered NICE’s thresholds of £20,000–£30,000 per QALY gained [22, 32, 33, 41]. Sweden studies showed a wider range for this threshold, from 200,000 SEK (€21,000) [29] to €55,000 per QALY gained [37]. The latter was very similar to the threshold applied in the German study (€50,000 per QALY gained) [28]. None of them was far from the US threshold’s commonly accepted standard of $50,000 per QALY gained.

### Limitations of the systematic review

There are several limitations that may affect our review findings. First, we cannot be sure that no relevant study is missing from this systematic review. However, in order to find as many relevant studies as possible, we have performed the search in PubMed and EMBASE, the most comprehensive databases in health sciences, as recommended [43], as well as in a specific database for economic evaluations. In addition, we designed a very sensitive search strategy (yielding the 8,789 titles revised) and we performed an additional manual reference search. Second, no quantitative synthesis of the results by meta-analysis was planned due to the well-known high heterogeneity among health economic evaluations. Furthermore, considering the scarce number of studies comparing the same interventions, obtaining a pooled estimator would make no sense. Third, internal validity of the synthesis provided by a systematic review depends on the quality of primary studies. In our systematic review, quality could be considered good except for effectiveness, which failed in almost half of the studies. It is necessary to take into account that recruitment for randomized trials presented considerable difficulties in these patients [44, 45], and the only available trial, the SPCG-4 [40]–which was used in several of these economic evaluations, was conducted at the beginning of PSA era. Fourth, studies with a cost-comparison design were included despite not being economic evaluations. However, the information they provided clearly contributed to the amount and robustness of evidence on costs. Finally, Fig. 2 shows reported direct healthcare costs without transforming them into a single year to avoid manipulation. We only converted currency into euros, using 2015 exchange rates, to facilitate comparisons.

## Conclusions

To our knowledge, this is the first systematic literature review of the European economic evaluations of all main primary treatments for localized prostate cancer published during the last 15 years. The 13 studies identified (five comparing interventions with expectant management, four contrasting robotic with non-robotic assisted surgery, three assessing new modalities of radiotherapy, and three comparing radical prostatectomy with brachytherapy) showed that currently relevant treatment alternatives for localized prostate cancer are scarcely assessed in economic evaluations in the European countries. Furthermore, differences between cost-comparison and cost-effectiveness studies suggest underestimation of costs in studies based on models from theoretical cohorts.

In conclusion, very limited evidence supports the cost-effectiveness of radical prostatectomy versus watchful waiting, and that of brachytherapy versus radical prostatectomy. Regarding the evaluation of new treatment modalities, also limited evidence supports the cost-effectiveness of robotic-assisted laparoscopic radical prostatectomy versus non-robotic procedures, and that of brachytherapy, IMRT and proton therapy versus traditional external radiotherapy. Relevant disparities were detected among studies, mainly based on effectiveness. These apparently contradictory results may be reflecting the difficulty of interpreting small differences between treatments regarding QALY gains. Moreover, despite an acceptable methodological quality in most aspects of the studies included, the effectiveness uncertainty could jeopardize the internal validity of their results.

## Additional files

Additional file 1: MEDLINE, EMBASE and NHS EED (NHS Economic Evaluation Database, CRD York) specific search strategies. (DOC 74 kb) Additional file 2: Patient Intervention Comparator Outcome (PICO) strategy. (DOC 33 kb)

## Abbreviations

2DRT: Two Dimensional Radiotherapy
3DCRT: Three Dimensional Conformal Radiation Therapy
AS: Active Surveillance
BT: Brachytherapy
DRG: Diagnosis Related Group
ERT: External Radiation Therapy
EM: Expectant Management
ICERs: Incremental Cost-Effectiveness Ratios
IMRT: Intensity-Modulated Radiation Therapy
LE: Life Expectancy
LRP: Laparoscopic Prostatectomy
NHS: British National Health System
NHS EED: NHS Economic Evaluation Database
NICE: National Institute for Clinical Excellence
PICO: Patient Intervention Comparator Outcome
PRISMA: Preferred Reporting Items for Systematic Reviews and Meta-Analyses
PRP: Perineal Radical Prostatectomy
PSA: Prostate-Specific Antigen
QALY: Quality-Adjusted Life-Year
RALP: Robot-Assisted Laparoscopic Prostatectomy
RP: Radical Prostatectomy
RRP: Radical Retropubic Prostatectomy
SPCG-4: Scandinavian Prostatic Cancer Group Study Number 4
UK: United Kingdom
US: United States
WW: Watchful Waiting
