# Supplementary material for: Economic evaluation of treatments for patients with localized prostate cancer in Europe: a systematic review
Source: BMC Health Serv Res. 2016 Oct 3;16:541. doi: 10.1186/s12913-016-1781-z (PMC5048403; doi:10.1186/s12913-016-1781-z)
Supplement: Additional file 1: — MEDLINE, EMBASE and NHS EED (NHS Economic Evaluation Database, CRD York) specific search strategies. (DOC 74 kb) [file 12913_2016_1781_MOESM1_ESM.doc]

Supplementary information 1.

**MEDLINE, EMBASE and NHS EED (NHS Economic Evaluation Database, CRD York) specific search strategies.**

**A. MEDLINE**

| **Search term** |
| --- |
| 1. (((((((("Hospital Costs"[Mesh] OR "Costs and Cost Analysis"[Mesh] OR "Employer Health Costs"[Mesh] OR "Health Care Costs"[Mesh] OR "Drug Costs"[Mesh] OR "Direct Service  Costs"[Mesh] OR "Cost of Illness"[Mesh] OR "Cost-Benefit  Analysis"[Mesh] OR "Economics"[Mesh]) OR ("economics"[Subheading] OR "economics"[All Fields] OR  "economics"[MeSH Terms])) OR ("costs and cost analysis"[MeSH  Terms] OR ("costs"[All Fields] AND "cost"[All Fields] AND "analysis"[All Fields]) OR "costs and cost analysis"[All Fields] OR  ("cost"[All Fields] AND "analysis"[All Fields]) OR "cost  analysis"[All Fields])) OR ("economics"[Subheading] OR "economics"[All Fields] OR "fees"[All Fields] OR "fees and charges"[MeSH Terms] OR ("fees"[All Fields] AND "charges"[All Fields]) OR "fees and charges"[All Fields])) OR ("fees and charges"[MeSH Terms] OR ("fees"[All Fields] AND "charges"[All Fields]) OR "fees and charges"[All Fields] OR "charge"[All Fields])) OR ("hospital charges"[MeSH Terms] OR ("hospital"[All Fields] AND "charges"[All Fields]) OR "hospital charges"[All Fields])) OR ("budgets"[MeSH Terms] OR "budgets"[All Fields] OR "budget"[All Fields])) OR ("commerce"[MeSH Terms] OR "commerce"[All Fields] OR "price"[All Fields])) OR ("economics"[Subheading] OR "economics"[All Fields] OR "cost"[All Fields] OR "costs and cost analysis"[MeSH Terms] OR ("costs"[All Fields] AND "cost"[All Fields] AND "analysis"[All Fields]) OR "costs and cost analysis"[All Fields])) |
| 2. ((((((("prostatic neoplasms"[MeSH Terms] OR ("prostatic"[All  Fields] AND "neoplasms"[All Fields]) OR "prostatic neoplasms"[All Fields]) OR ("prostate"[MeSH Terms] OR  "prostate"[All Fields] OR "prostatic"[All Fields])) OR  ("prostate"[MeSH Terms] OR "prostate"[All Fields])) OR (("prostate"[MeSH Terms] OR "prostate"[All Fields] OR "prostatic"[All Fields]) AND ("carcinoma"[MeSH Terms] OR "carcinoma"[All Fields]))) OR ("prostatic neoplasms"[MeSH Terms] OR ("prostatic"[All Fields] AND "neoplasms"[All Fields]) OR "prostatic neoplasms"[All Fields] OR ("prostate"[All Fields] AND "cancer"[All Fields]) OR "prostate cancer"[All Fields])) OR ("prostatic neoplasms"[MeSH Terms] OR ("prostatic"[All Fields] AND "neoplasms"[All Fields]) OR "prostatic neoplasms"[All Fields] OR ("prostatic"[All Fields] AND "cancers"[All Fields]) OR "prostatic cancers"[All Fields])) OR ("prostatic hyperplasia"[MeSH Terms] OR ("prostatic"[All Fields] AND "hyperplasia"[All Fields]) OR "prostatic hyperplasia"[All Fields] OR ("prostatic"[All Fields] AND "adenoma"[All Fields]) OR "prostatic adenoma"[All Fields]))) NOT ("prostatic hyperplasia"[MeSH Terms] OR ("prostatic"[All Fields] AND "hyperplasia"[All Fields]) OR "prostatic hyperplasia"[All Fields] OR ("benign"[All Fields] AND "prostate"[All Fields] AND "hyperplasia"[All Fields]) OR "benign prostate hyperplasia"[All Fields]) NOT "Prostatic Hyperplasia"[Mesh] AND ("2000/01/01"[PDAT] : "2015/12/31"[PDAT]) |
| 3. 2 AND 3 |

**B. EMBASE**

| **Search term** |
| --- |
| 1. ('socioeconomics'/exp or 'cost benefit analysis'/exp or 'cost effectiveness analysis'/exp or 'cost of illness'/exp or 'cost  control'/exp or 'economic aspect'/exp or 'financial management'/exp or 'health care cost'/exp or 'health care  financing'/exp or 'health economics'/exp or 'hospital cost'/exp or  (fiscal:ab,ti or financial:ab,ti or finance:ab,ti or funding:ab,ti) or  'cost minimization analysis'/exp or (cost$ and estimate$) or (cost$  and variable$) or (unit and cost$)) |
| 2. (('prostate cancer' or 'prostatic neoplasms' or (prostate:ab,ti  and cancer:ab,ti) or (prostatic:ab,ti and cancer:ab,ti) or (prostat:ab,ti and cancer:ab,ti) or (prostate:ab,ti and carcinoma:ab,ti) or (prostatic:ab,ti and carcinoma:ab,ti) or (prostat:ab,ti and carcinoma:ab,ti)) |
| 3. ('prostate hypertrophy' or (prostate:ab,ti and hyperplasia:ab,ti) or (prostatic:ab,ti and hyperplasia:ab,ti) or (prostat:ab,ti and hyperplasia:ab,ti))) |
| 4. 2 NOT 3 |
| 5. 1 AND 4 |
| 6. #5 AND [embase]/lim |
| 7. #5 AND [embase]/lim AND [2000-2015]/py |
| 8. #5 AND [embase]/lim AND [2000-2015]/py AND [humans]/lim |

**C. NHS EED (NHS Economic Evaluation Database, CRD York)**

| **Search term** |
| --- |
| 1. Prostate AND Cancer |
