# Supplementary material for: Economic evaluation of treatments for patients with localized prostate cancer in Europe: a systematic review
Source: BMC Health Serv Res. 2016 Oct 3;16:541. doi: 10.1186/s12913-016-1781-z (PMC5048403; doi:10.1186/s12913-016-1781-z)
Supplement: Additional file 2: — Patient Intervention Comparator Outcome (PICO) strategy. (DOC 33 kb) [file 12913_2016_1781_MOESM2_ESM.doc]

Additional file 2.

**Patient Intervention Comparator Outcome (PICO) strategy.**

| **Criteria** |  |
| --- | --- |
| Population | Men with localized prostate cancer  If unclear: include participants stated to have prostate cancer, or prostate related diseases |
| Intervention | Treatments for localized prostate cancer:  Radical prostatectomy OR External Radiotherapy OR Brachytherapy  Specific interventions for prostate cancer may not be reported in the abstract |
| Comparators | Any treatment for stated Interventions  No treatment |
| Outcomes | Do not exclude on outcomes at abstract screening stage.  At full-text screening: outcomes include:  Incremental cost per quality-adjusted life-year (primary outcome) Incremental cost-effectiveness ratios (ICERs)  Other measures of cost-effectiveness  Costs comparisons |
| Timepoints/ follow-up | Any |
| Study type | Cost-effectiveness, cost-benefit or cost-utility studies  Comparative studies  If unclear: include studies reporting costs or resource use in this population |
| Publication date | January 2000– March 2015 |
| Publication language | Any European language |
| Setting | Any European country |
